# Unusual Clinical Course of Odontoid Fracture: Transient Prehospital Cardiopulmonary Arrest

**DOI:** 10.7759/cureus.12157

**Published:** 2020-12-18

**Authors:** Keisuke Maeda, Toshihisa Ichiba

**Affiliations:** 1 Department of Emergency Medicine, Hiroshima Citizens Hospital, Hiroshima, JPN

**Keywords:** odontoid fracture, neurogenic shock, cardiopulmonary arrest

## Abstract

Odontoid fracture is the most common type of cervical spine fracture in the elderly. Neurological injury due to odontoid fracture is uncommon, but if the injury is severe, it can lead to cardiac arrest. We present a case of odontoid fracture with transient cardiac arrest just after the fall, which fully recovered in a few minutes before arrival at the hospital.

A 62-year-old man fell down on a street, and compressions were performed by a witness. On arrival of the emergency medical service, he showed pulseless electrical activity. After two minutes of cardiac resuscitation, he experienced a return of spontaneous circulation and was breathing spontaneously. On arrival at our hospital, his blood pressure was 171/106 mmHg, heart rate was 100 beats per minute, and respiratory rate was 12 times per minute, but he was at Glasgow Coma Scale 3 with an alcohol odor from exhaled breath. Six hours after admission, his level of consciousness improved, and he complained of neck pain and difficulty in movement of his arms and legs. CT revealed a fracture and posteriorly displaced C2 bone. MRI showed a hyper-intense area from C1 to C2. We made a diagnosis of spinal cord injury caused by an odontoid fracture that led to cardiac arrest.

An odontoid fracture can cause transient cardiac arrest just after a fall. The possibility of odontoid fracture associated cervical spine injury should be considered in elderly and unconscious patients with minor trauma. Early CT cervical spine in selected patients can be helpful, especially in patients with cardiac arrest, even if it lasted for only a short prehospital period.

## Introduction

Odontoid fracture is a common cervical spine fracture of the C2 vertebra in the elderly. It usually results from low-energy impacts such as ground-level falls with an associated forced hyperextension mechanism [1,2]. Typical presentation in patients with an odontoid fracture is neck pain, and accompanying neurological injury is uncommon. However, when severe injury occurs, it can lead to cardiac arrest (CA) because of the high level of cervical spinal cord injury [3]. We present a case of odontoid fracture with transient CA just after a fall, with a return of spontaneous circulation (ROSC) in a few minutes before arrival at the hospital.

## Case presentation

**Figure 1:**
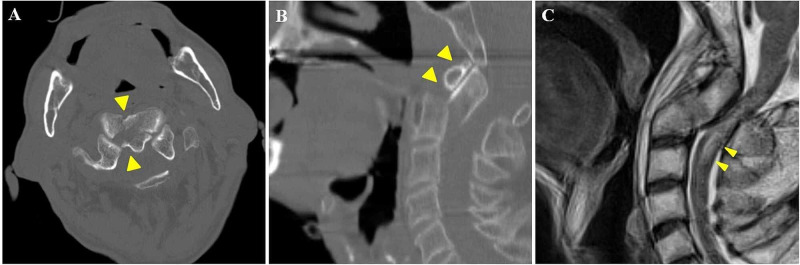
(A) CT revealed a fracture of the C2 vertebra (arrowhead). (B) Sagittal reconstruction of the CT images revealed a posteriorly displaced bone fragment (arrowhead). (C) Sagittal T2-weighted MRI of the neck showed a hyper-intensity area from C1 to C2 (arrowhead).

A 62-year-old man fell down on a street, and compressions were performed by a witness. He did not take steroids or medications for osteoporosis, nor did he take anticoagulants. When the emergency medical service (EMS) arrived, his heart rate was 30 beats per minute without palpability, and breathing was agonal gasping, indicating pulseless electrical activity. After two minutes of cardiopulmonary resuscitation without medications or tracheal intubation, he experienced ROSC and was breathing spontaneously. He was then transported to our hospital, which took 20 minutes. On arrival at our hospital, his blood pressure was 171/106 mmHg, heart rate was 100 beats per minute, and respiratory rate was 12 times per minute with a normal breathing pattern. Neurological findings indicated a Glasgow Coma Scale (GCS) score of 3 with an alcohol odor from exhaled breath, pupil 4 mm with no difference between left and right, and normal reflection. He had a scratch on the front of his head. Whole-body computed tomography (CT) and magnetic resonance imaging (MRI) of the head showed no findings that could cause disturbance of consciousness. His blood glucose level was 119 mg/dL, and other laboratory tests also showed no abnormalities that could cause CA. Electrocardiogram and echocardiography also showed no abnormal findings, such as ST elevation or ventricular asynergy, suggesting cardiogenic CA. In addition, his consciousness had improved to GCS 10 during initial care in the emergency room, so we did not perform targeted temperature management. Then he was admitted with an unexplained CA and consciousness disorder. Six hours after admission, his level of consciousness completely improved. He complained of neck pain and difficulty in movement of his arms and legs, which was consistent with the American Spinal Injury Association (ASIA) impairment scale D. We, therefore, reconstructed the CT images and reevaluated them. The CT images revealed a fracture of the C2 vertebra, and sagittal reconstruction images revealed a posteriorly displaced bone fragment without retropharyngeal hematoma (Figures 1A, 1B). Sagittal T2-weighted MRI of the neck was performed and showed a hyper-intense area from C1 to C2 (Figure 1C). According to these findings, we made a diagnosis of incomplete spinal cord injury at the C1 to C2 level caused by an odontoid fracture that led to transient CA. We initially treated the patient nonoperatively with the application of a halo jacket. However, the odontoid fragment was displaced again, and we performed an operation with posterior fixation of the C1-2 vertebra on the 43rd hospital day. After the operation, he was able to walk with a walker and was discharged for rehabilitation.

## Discussion

The course of the patient indicated two important clinical issues: 1) an odontoid fracture can cause transient CA just after a fall, and 2) a diagnosis of cervical spine injury might be missed in patients with impaired consciousness.

There are two possible causes for this patient's shock and CA. One is neurogenic, and the other is respiratory shock and CA due to spinal compression. Neurologic injury in patients with an odontoid fracture is uncommon, but most patients with CA before arrival at hospital die despite cardiopulmonary resuscitation [4]. In our case, vital signs initially showed pulseless electrical activity, but on arrival, the vital signs returned to normal. As a result, we were initially not aware of spinal cord injury. However, the patient was resuscitated and maintained without adrenaline, which was unusual for neurogenic shock, so the likelihood of neurogenic shock and CA may be lower. The other possible cause was respiratory shock and CA due to spinal cord compressions. We speculated that pressure of the displaced odontoid fracture and severe stenosis caused by hyperextension of the head caused transient serious spinal cord injury and led to transient respiratory arrest and CA and that basic cardiopulmonary resuscitation and return to a normal head position relieved the stenosis of the spinal canal, leading to recovery from respiratory arrest before arrival at the hospital [5]. The patient’s clinical course indicated that a spinal compression caused by odontoid fracture could cause transient neurologic or respiratory shock od CA just after a fall. Therefore, we should know this rare clinical course of an odontoid fracture and spinal compression and pay attention to vital signs not only on arrival at the hospital but also on prehospital first patient contact.

The incidence of delayed diagnosis of cervical spine injuries has been reported to be about 5% [6]. Radiologic misinterpretation, trauma patients with multiple injuries, and impaired consciousness are common reasons for delayed diagnosis [7]. Our patient was also unconscious on arrival at the hospital because of alcohol consumption and transient CA, which resulted in a delayed diagnosis of the odontoid fracture. However, thinking back later, the scratch on the front of his head, the episode of falling, and the vital signs mentioned above suggested hyperextension of the head and spinal cord injury. We should perform CT of the cervical spine in patients who cannot be assessed for symptoms or signs and examine with sagittal and coronal reconstruction images [7].

## Conclusions

Early detection of cervical spine injury is essential because delayed diagnosis might lead to poor consequences. The possibility of odontoid fracture-associated cervical spine injury should be considered in elderly and unconscious patients with minor trauma. Early CT cervical spine in selected patients can be helpful, especially in patients with neurogenic shock or CA, even if it lasted for only a short prehospital period.
